# Topological machine learning for protein–nucleic acid binding affinity changes upon mutation

**DOI:** 10.1088/2632-2153/ae1714

**Published:** 2025-11-05

**Authors:** Xiang Liu, Junjie Wee, Guo-Wei Wei

**Affiliations:** 1 Department of Mathematics, Michigan State University, East Lansing, MI 48824, United States of America; 2 Department of Electrical and Computer Engineering, Michigan State University, East Lansing, MI 48824, United States of America; 3 Department of Biochemistry and Molecular Biology, Michigan State University, East Lansing, MI 48824, United States of America

**Keywords:** protein–DNA, protein–RNA, binding affinity changes upon mutation, persistent Laplacians, topological machine learning

## Abstract

Understanding how protein mutations affect protein–nucleic acid binding is critical for unraveling disease mechanisms and advancing therapies. Current experimental approaches are laborious, and computational methods remain limited in accuracy. To address this challenge, we propose a novel topological machine learning model (TopoML) combining persistent Laplacian (from topological data analysis) with multi-perspective features: physicochemical properties, topological structures, and protein Transformer-derived sequence embeddings. This integrative framework captures robust representations of protein–nucleic acid binding interactions. To validate the proposed method, we employ two datasets, a protein–DNA dataset with 596 single-point amino acid mutations, and a protein–RNA dataset with 710 single-point amino acid mutations. We show that the proposed TopoML model outperforms state-of-the-art methods in predicting mutation-induced binding affinity changes for protein–DNA and protein–RNA complexes.

## 1. Introduction

Protein–nucleic acid interactions play fundamental roles in a variety of biological activities, including DNA replication, transcription, and translation [1-3]. Since nucleic acids are highly charged polymers, protein–nucleic acid interactions are largely determined by various intermolecular polar forces, such as hydrogen bonding, dipolar forces, electrostatic interactions, although van der Waals forces and hydrophobic interactions may play a minor role. Even a single amino acid mutation in a protein, such as from a positively charged amino acid residue to a negatively charged one, can disrupt these forces, thereby altering the interaction with nucleic acids [4, 5]. This may result in significant functional impairments, leading to various diseases [6-9]. Therefore, investigating the impact of amino acid mutations on protein–nucleic acid interactions is critical for understanding disease mechanisms and for guiding the development of effective therapeutic interventions.

The strength of protein–nucleic acid interactions is typically quantified by binding affinity (ΔG) or dissociation constant (*K*_d_). The binding affinity change from a mutation is commonly computed as ΔΔG=ΔG(mutant)−ΔG(wild-type). Several experimental methods, including surface plasmon resonance [10], isothermal titration calorimetry [11], and fluorescence resonance energy transfer [12], have been developed to measure the binding affinities between proteins and nucleic acids. However, these experimental methods are often time-consuming and labor-intensive, making them unsuitable for large-scale mutational analysis. Consequently, there is a critical need to develop computational approaches for predicting protein–nucleic acid binding affinity changes upon mutation.

Over the past decade, numerous machine learning models have been proposed to predict the protein–nucleic acid binding affinity changes upon mutation. In general, these models aim to learn a function f:PN→ΔΔG, where the input consists of the protein–nucleic acid complex together with mutation information, and the output corresponds to the predicted change in binding free energy. Among these, SAMPDI [13], SAMPDI-3D [14], SAMPDI-3Dv2 [15], and PremPDI [16] focus on protein–DNA interactions, while PremPRI [17] and PRA-MutPred [18] are designed for protein–RNA interactions. Some models, such as mCSM-NA [19], PEMPNI [20], and PNBACE [21], are for both protein–DNA and protein–RNA interactions. SAMPDI-3Dv2 and PRA-MutPred represent the most recent advancements for protein–DNA and protein–RNA interactions, respectively. The S596 dataset provided by SAMPDI-3Dv2 and the dataset of 710 mutations provided by PRA-MutPred are currently the largest available protein–DNA and protein–RNA datasets that have been used for evaluating models in the prediction of mutation-induced binding affinity changes. Despite these developments, the performance of these models remains modest, with SAMPDI-3Dv2 and PRA-MutPred achieving Pearson correlation coefficient (PCCs) of only 0.65 and 0.70 in the *k*-fold cross-validation, respectively, calling for novel strategies to enhance prediction accuracy.

Topological data analysis (TDA) is an emerging field that uses topological tools, particularly, persistent homology, to analyze the shape and structural patterns within data [22, 23]. In computational biology, TDA was combined with machine learning for protein classification [24]. Topological deep learning (TDL) was first introduced by Cang and Wei in 2017 to integrate TDA and deep neural networks [25]. In the same work, TDL outperformed other competing approaches in protein–ligand binding and protein mutation predictions [25]. TDL has become the new frontier in rational learning [26]. Most impressively, this approach achieved victories in D3R Grand Challenges, a global competition series in computer-aided drug design [27, 28]. Essentially, TDA approaches are extremely effective for intrinsically complex multiscale data, such as those from the biomolecular structures. However, persistent homology has many limitations, including its insensitivity to no topological shape evolution. Persistent spectral graph, also known as persistent Laplacian, was introduced to overcome some limitations of persistent homology by Wang *et al* [29] Persistent Laplacians not only encode topological information through the harmonic spectra (i.e. persistent homology), but also capture rich geometric properties via non-harmonic spectra [30]. A fast algorithm was proposed [31], and stability analysis [32] was given to persistent Laplacians, which justifies successful applications, such as protein–ligand binding prediction [33]. Persistent Laplacians were shown to outperform persistent homology with over 30 datasets in protein engineering [34]. Notably, persistent Laplacians have enabled the early prediction of emerging dominant SARS-CoV-2 variants, such as Omicron BA.4 and BA.5, two months prior to their announcement by the World Health Organization [35].

It is worth to mentioning that in addition to earlier TDL prediction of protein stability changes upon mutations [25], several TDA-based models, such as TopNetTree [36], PerSpect-EL [37], HCML [38], PTA [39], and TopNetmAb [35] have demonstrated strong performance in predicting mutation-induced protein–protein binding affinity changes. Despite the success of these approaches in protein–protein interaction studies, TDL or TDA-based methods, particularly those leveraging persistent Laplacians, have not been applied to protein–nucleic acid interactions.

In this work, we build a persistent-Laplacian-based topological machine learning (TopoML) model for predicting mutation-induced protein–nucleic acid binding affinity changes. The TopoML model integrates three distinct types of features: (1) topological features derived from persistent Laplacians, (2) physicochemical features, (3) sequence features by a pretrained protein Transformer. These three types of features are concatenated as the input for a gradient boosting tree algorithm to perform the prediction task. We evaluate TopoML on two benchmark datasets: the S596 dataset comprising 596 mutations for protein–DNA interactions, and a dataset of 710 mutations for protein–RNA interactions. The results demonstrate that TopoML outperforms other competing methods in the predictions of both protein–DNA and protein–RNA interactions, highlighting the effectiveness of incorporating topological features for mutation impact prediction.

## 2. Results

### 2.1. Overview of the TopoML model

**Figure 1.**
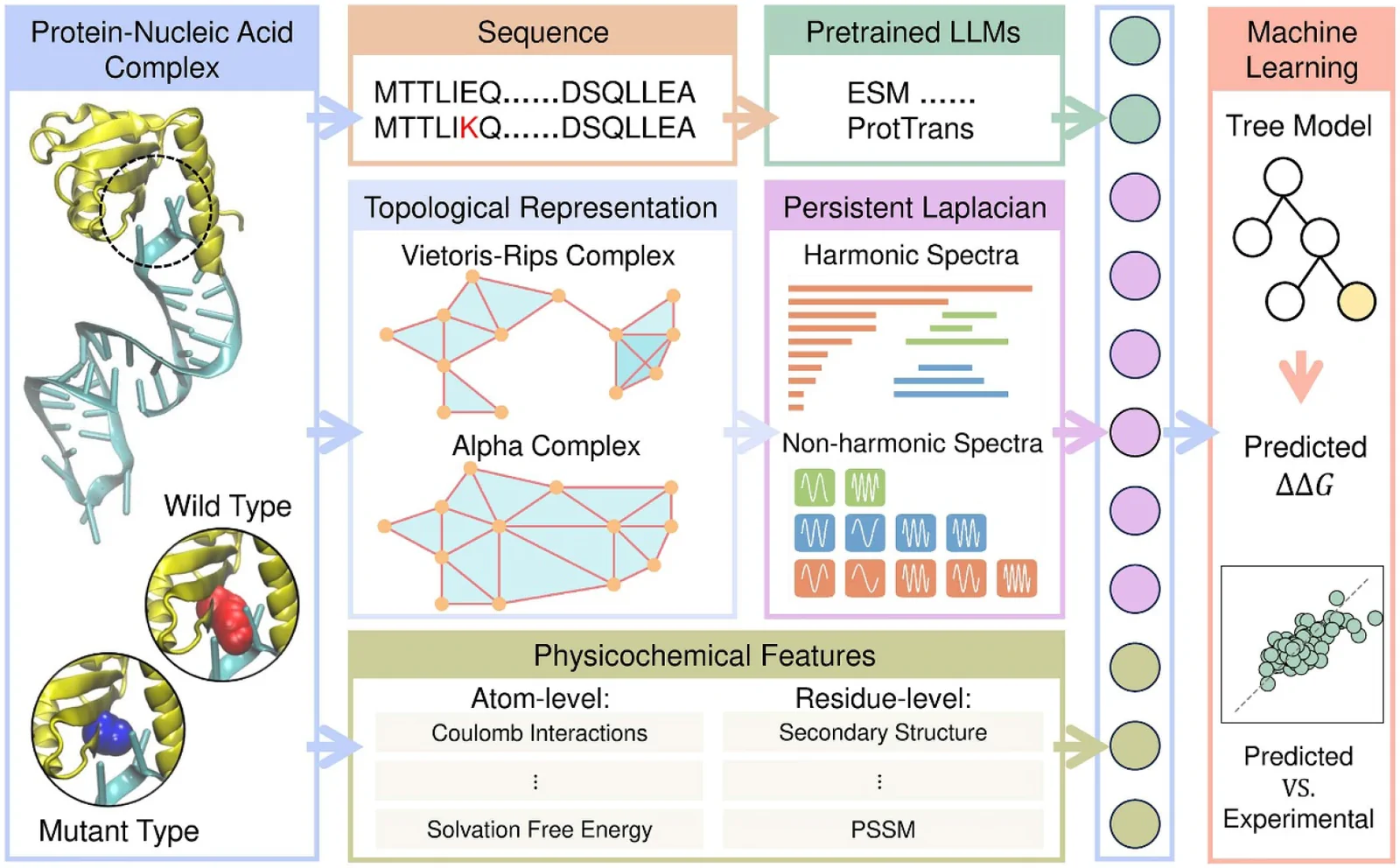
Illustration of TopoML model architecture. For each protein–nucleic acid complex, TopoML extracts a comprehensive set of features and employs a gradient boosting tree algorithm to predict mutation-induced binding affinity changes. The feature generation pipeline comprises three components: sequence features derived from the pretrained protein Transformer models, topological features computed via persistent Laplacian, and the physical and chemical features. These three sets of features are concatenated to form the input of the machine learning algorithm for the prediction task.

The overall workflow of our model is illustrated in figure 1. As a standard machine learning model, TopoML extracts features from protein–nucleic acid complexes and uses a gradient boosting tree algorithm to predict the protein–nucleic acid binding affinity changes upon mutation. The feature generation process consists of three components: sequence features from a pretrained protein Transformer, topological features from persistent Laplacians, and physicochemical features. For sequence features, the FASTA sequences of the wild-type and mutant proteins are extracted from the complex and input into the pretrained Transformer models. The derived latent space embeddings are used as the sequence features. For topological features, the binding site and mutation site of the protein–nucleic acid before and after mutation are represented as simplicial complexes, and their persistent Laplacians are computed to generate the features. The harmonic spectra of persistent Laplacians are exactly the persistent homology information that characterizes the topological structure information while the non-harmonic spectra encode more geometric information of the protein–nucleic complex. For physicochemical features, we consider the atom-level properties such as partial charge, electrostatic solvation free energy, and Coulomb interactions, as well as residue-level properties such as mutation site neighbor amino acid composition, p*K_a_* shifts, and additional physicochemical properties. These three types of feature embeddings are concatenated to form the input feature vector for the machine learning algorithm (gradient boosting tree [40]) to predict the mutation-induced binding affinity changes.

### 2.2. Evaluation of the model

#### 2.2.1. Datasets

Both protein–DNA and protein–RNA complexes are considered to study the effects of the amino acid mutations on binding affinities. For protein–DNA interactions, we use the S596 dataset introduced in a recent work [15]. This dataset was systematically curated from the ProNAB [41], ProNIT [42], and dbAMEPNI [43] databases, and contains 596 single-point amino acid mutations with experimentally measured ΔΔG. For protein–RNA interactions, we consider the dataset from PRA-MutPred [18], which was curated from the ProNAB database, and includes 710 single-point amino acid mutations with experimentally determined ΔΔG. These two datasets are, to the best of our knowledge, the largest known protein–DNA and protein–RNA datasets that have been used for evaluating models on the prediction of mutation-induced binding affinity changes.

#### 2.2.2. Evaluation protocols

PCC, mean absolute error (MAE), and root mean square error (RMSE) are employed as evaluation metrics to facilitate a fair comparison with benchmark methods reported in the literature [15, 17-21, 44]. To enhance the reliability of the results, we independently repeated the process 100 times and used the average value as the final performance of our model.

#### 2.2.3. Performance on protein–RNA interactions

For protein–RNA interactions, the dataset contains 710 single-point amino acid mutations from 141 protein–RNA complexes. Among these mutations, 595 are used as the training set, while the remaining 115 constitute the testing set. Following the strategy employed by PRA-MutPred [18], we expand the training set by incorporating reverse mutations, obtained by reversing the mutations and assigning the opposite ΔΔG values as labels. Consequently, we get a training set of 1190 mutations and a test set of 115 mutations.

##### Comparison with existing methods

**Figure 2.**
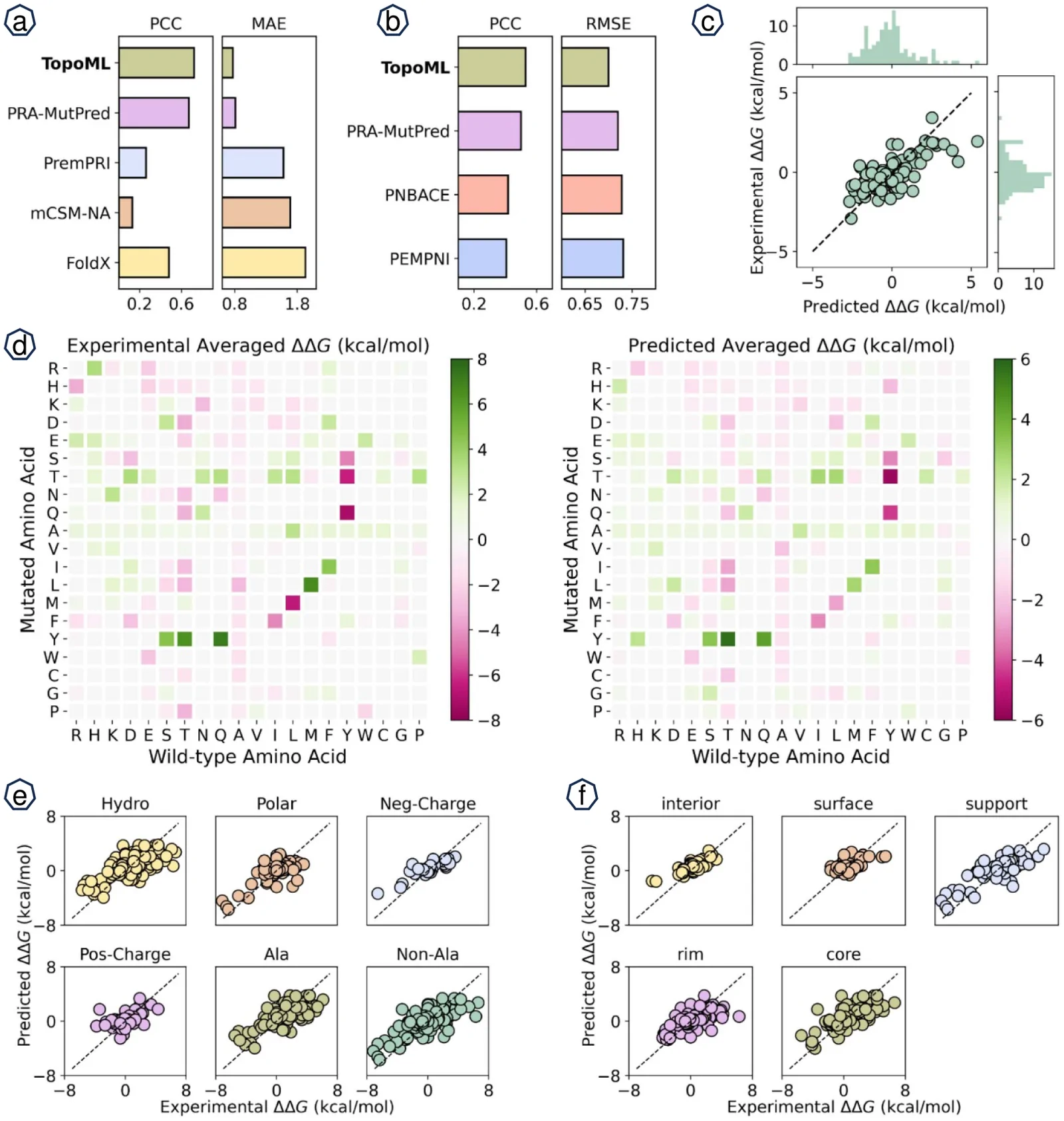
Illustration of model performance for predicting protein–RNA binding free energy changes upon mutation in the S710 dataset. (a) Performance comparison between our model and existing methods on the test set of 115 mutations. (b) Performance comparison on the MPR79 test set of 79 mutations, following the protocol used in PRA-MutPred. (c) Correlation between experimental and predicted ΔΔG values for the test set of 115 mutations. (d) Residue-residue matrix comparing the averaged experimental and predicted mutation-induced ΔΔG values for the whole dataset of 710 mutations. (e) Model performance across different mutation types based on residue physicochemical properties. (f) Model performance across different structural regions defined by relative accessible solvent area (rASA).

We evaluate the performance of our model in the test set and compare it with existing methods. The performance comparison is shown in Figure 2(a) Our model can achieve the best results. Specifically, the FoldX model yields a PCC of 0.48 and an MAE of 1.93 kcal mol^−1^. The mCSM-NA model achieves a PCC of 0.13 with an MAE of 1.69 kcal mol^−1^. The PremPRI model has a PCC of 0.26 and an MAE of 1.58 kcal mol^−1^. PRA-MutPred, the method originally constructed the dataset, reports a PCC of 0.67 and an MAE of 0.81 kcal mol^−1^. In contrast, our model achieves a PCC of 0.72 and an MAE of 0.77 kcal mol^−1^. A direct comparison between the experimental and predicted ΔΔG values of our model in the test set is shown in figure 2(c).

In addition to these models, we also compare our method with PEMPNI, an energy-based model, and its updated version, PNBACE, both of which are designed to predict protein–nucleic acid binding affinity changes upon mutation. These models rely on computationally intensive energy-based features. Following the evaluation strategy of PRA-MutPred, we adopt the test set MPR79, used in the PNBACE study, for a fair comparison, and remove any overlapping mutations from our training set to avoid data leakage. Our model is trained on the remaining data and evaluated on the MPR79 test set. The performance comparison is shown in figure 2(b). Our model continues to outperform all baselines. Specifically, PEMPNI has a PCC of 0.41 and an RMSE of 0.73 kcal mol^−1^, while PNBACE reports a PCC of 0.42 with an RMSE of 0.73 kcal mol^−1^. The PRA-MutPred model obtains a PCC of 0.50 and an RMSE of 0.72 kcal mol^−1^. In contrast, our model achieves the best performance with a PCC of 0.53 and an RMSE of 0.70 kcal mol^−1^.

To further evaluate the robustness of our model, we conduct a random 10-fold cross-validation on the full set of 710 mutations. Our model achieves an average PCC of 0.721 and an MAE of 0.785 kcal mol^−1^, outperforming PRA-MutPred, which reports a PCC of 0.70 and an MAE of 0.90 kcal mol^−1^ under the same setting.

##### Performance analysis based on mutation types

Using the predictions from the 10-fold cross-validation, we compare the average experimental and our predicted ΔΔG values across different mutation types. As shown in figure 2(d), a residue-residue matrix is used where the *x*-axis represents the wild-type residues and the *y*-axis represents the mutated residues. The entry at row of residue ‘−’ and column of residue ‘+’ is the average ΔΔG of all mutations with wild type ‘+’ and mutant type ‘−’. In the matrix representation, the reverse mutation of each mutation is also considered, the ΔΔG for the reverse mutation, i.e. from mutated type to wild-type, is set to be the opposite value. Consequently, the residue-residue matrix is antisymmetric. It can be seen that the predicted matrix closely mirrors the patterns observed in the experimental data, indicating that our model effectively captures mutation-type-specific trends in binding affinity changes. Although our model shares a similar pattern in average binding affinity changes compared to the experimental data, the variance of its predictions is generally lower, which requires further investigation. One interesting observation is that all mutations to alanine exhibit positive ΔΔG values. A possible explanation is that alanine, with its small and non-polar side chain, is often unable to compensate for the loss of favorable interactions, such as hydrogen bonds and electrostatic contacts that are originally contributed by larger or charged residues. This leads to a reduction in binding affinity and consequently results in positive ΔΔG values.

Furthermore, we group the mutation residues into different categories: hydrophobic, polar, negatively-charged, positively-charged, alanine, and non-alanine. The comparison between predicted values and experimental ones for each category is shown in figure 2(e). Specifically, the PCC (MAE) values are 0.716 (0.728 kcal mol^−1^), 0.73 (0.943 kcal mol^−1^), 0.87 (0.957 kcal mol^−1^), 0.469 (1.0 kcal mol^−1^), 0.709 (0.665 kcal mol^−1^), and 0.713 (1.0 kcal mol^−1^) for hydrophobic, polar, negative-charged, positive-charged, alanine, and non-alanine, respectively. Note that consistent results are achieved except the mutation type of positively-charged, which demonstrates our model’s ability to generalize across diverse amino acid substitutions without strong bias. The relatively lower performance observed for positively-charged mutations may be attributed to the small number of samples in this group, which includes only 57 mutations. This limited data size may reduce the model’s ability to capture reliable patterns for this category.

##### Performance analysis based on mutation regions

We group the mutation sites into five structural regions: interior, surface, support, rim, and core. These regions are determined by their relative accessible solvent area (rASA) using specific cutoff values [45] as detailed in table 1 of the supplementary information. Among these regions, mutations occurring in the core exhibit the highest average experimental ΔΔG value of 0.995 kcal mol^−1^, suggesting a greater destabilizing effect upon mutation in this buried region. The comparison between experimental energy changes and predicted ones across these regions is shown in figure 2(f). The corresponding PCC and MAE values are as follows: 0.83 (0.594 kcal mol^−1^) for interior, 0.484 (0.677 kcal mol^−1^) for surface, 0.815 (0.885 kcal mol^−1^) for support, 0.613 (0.83 kcal mol^−1^) for rim, and 0.727 (0.79 kcal mol^−1^) for core regions. The interior and support regions have the highest PCC, while the surface region has the lowest PCC. A possible explanation for the lower correlation in the surface region is the limited variability in its experimental data, which exhibits the smallest variance in ΔΔG values (1.05 kcal^−2^ mol^−2^). This reduced variance may hinder the model’s ability to capture meaningful trends, thereby decreasing correlation despite reasonable error magnitudes.

#### 2.2.4. Performance on protein–DNA interactions

For protein–DNA interactions, the S596 dataset is derived by combining the S419 and S117 datasets. Specifically, S419 was collected by the authors of the SAMPDI-3D model, while S117 is a newly curated dataset by the updated SAMPDI-3Dv2 model. The SAMPDI-3Dv2 model was trained on the S417 datset and evaluated on the S117 dataset, achieving a PCC of 0.17 and an RMSE of 1.34 kcal mol^−1^. Following the same training and testing strategy, our model TopoML achieves a superior performance with a PCC of 0.332 and an RMSE of 1.23 kcal mol^−1^. SAMPDI-3Dv2 also performed the 5-fold cross-validation on the S569 dataset, it obtained a PCC of 0.65 without an MAE reported. Under the same setting, our model achieved a PCC of 0.67 with an MAE of 0.61 kcal mol^−1^. To obtain more robust and unbiased results, we further conducted 10-fold cross-validation, which yielded an improved PCC of 0.681 and an MAE of 0.612 kcal mol^−1^.

**Figure 3.**
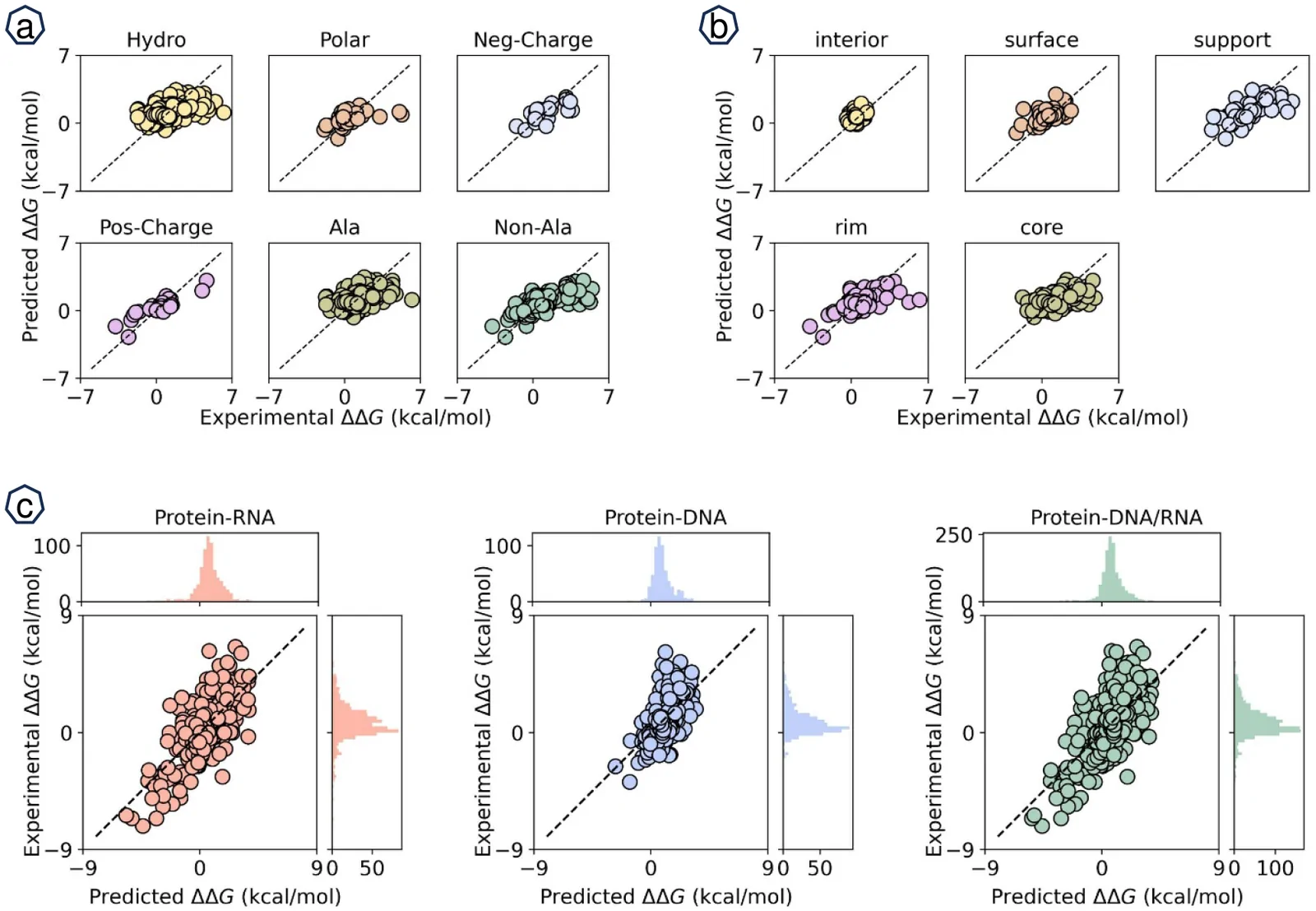
Illustration of model performance on predicting mutation-induced protein–DNA binding free energy changes for the S596 dataset. (a) Model performance across different mutation types based on residue physicochemical properties. (b) Model performance across different structural regions defined by relative accessible solvent area (rASA). (c) Comparison between experimental and predicted ΔΔG for protein–DNA interactions, protein–RNA interactions, and both of them.

Furthermore, we analyze the model performance across different mutation types based on the predictions from 10-fold cross-validation. The results are shown in figure 3(a). Specifically, the PCC (MAE) values are 0.631 (0.597 kcal mol^−1^) for hydrophobic, 0.498 (0.700 kcal mol^−1^) for polar, 0.745 (0.644 kcal mol^−1^) for negatively charged, 0.877 (0.648 kcal mol^−1^) for positively charged, 0.643 (0.582 kcal mol^−1^) for alanine, and 0.693 (0.673 kcal mol^−1^) for non-alanine mutations. Additionally, we evaluated model performance across different mutation regions, as shown in figure 3(b). The PCC and MAE values are 0.334 (0.477 kcal mol^−1^), 0.617 (0.481 kcal mol^−1^), 0.684 (0.812 kcal mol^−1^), 0.685 (0.592 kcal mol^−1^), and 0.638 (0.634 kcal mol^−1^) for interior, surface, support, rim, and core regions, respectively. The performance is consistent across most regions, except for the interior, where lower predictive accuracy may be attributed to the smaller variance of experimental ΔΔG values (variance of only 0.241 kcal^−2^ mol^−2^). Note that for protein–DNA interactions, our model achieves a PCC of 0.681 and an MAE of 0.876 kcal mol^−1^ using 10-fold cross-validation. When combining the 596 protein–DNA and 710 protein–RNA mutations into a unified dataset of 1306 mutations, our model achieves a PCC of 0.712 and an MAE of 0.708 kcal mol^−1^, further demonstrating the robustness of our model across interaction types. A comparison between the experimental and predicted ΔΔG values is shown in figure 3(c).

#### 2.2.5. Feature analysis

In our TopoML model, we incorporate three types of features: topological features derived from persistent Laplacians, sequence features obtained from a pretrained Transformer model, and physicochemical features. To evaluate the contribution of each feature type, we construct several ablation models using different combinations of these features. We denote the topological, sequence, and physicochemical features as Topo, Seq, and Phy, respectively. To ensure the reliability of the results, we perform 10-fold cross-validation on both the protein–DNA and protein–RNA interaction datasets. The performance of each feature combination is summarized in table 1.

For protein–DNA interactions, the topological features yield the best performance, achieving a PCC of 0.648 and an MAE of 0.65 kcal mol^−1^. In comparison, models using only physicochemical and sequence features achieve PCCs of 0.638 (MAE: 0.654 kcal mol^−1^) and 0.608 (MAE: 0.657 kcal mol^−1^), respectively. For protein–RNA interactions, all three feature types result in similar performance levels. These findings highlight the importance of topological features in capturing essential information, especially for protein–DNA interactions. Moreover, integrating all three feature types leads to a more robust and generalizable model performance. Recent studies have demonstrated that integrating multimodal protein information from both sequence and structure can enhance protein representation learning [46, 47]. Our findings are consistent with these observations.

**Figure 4.**
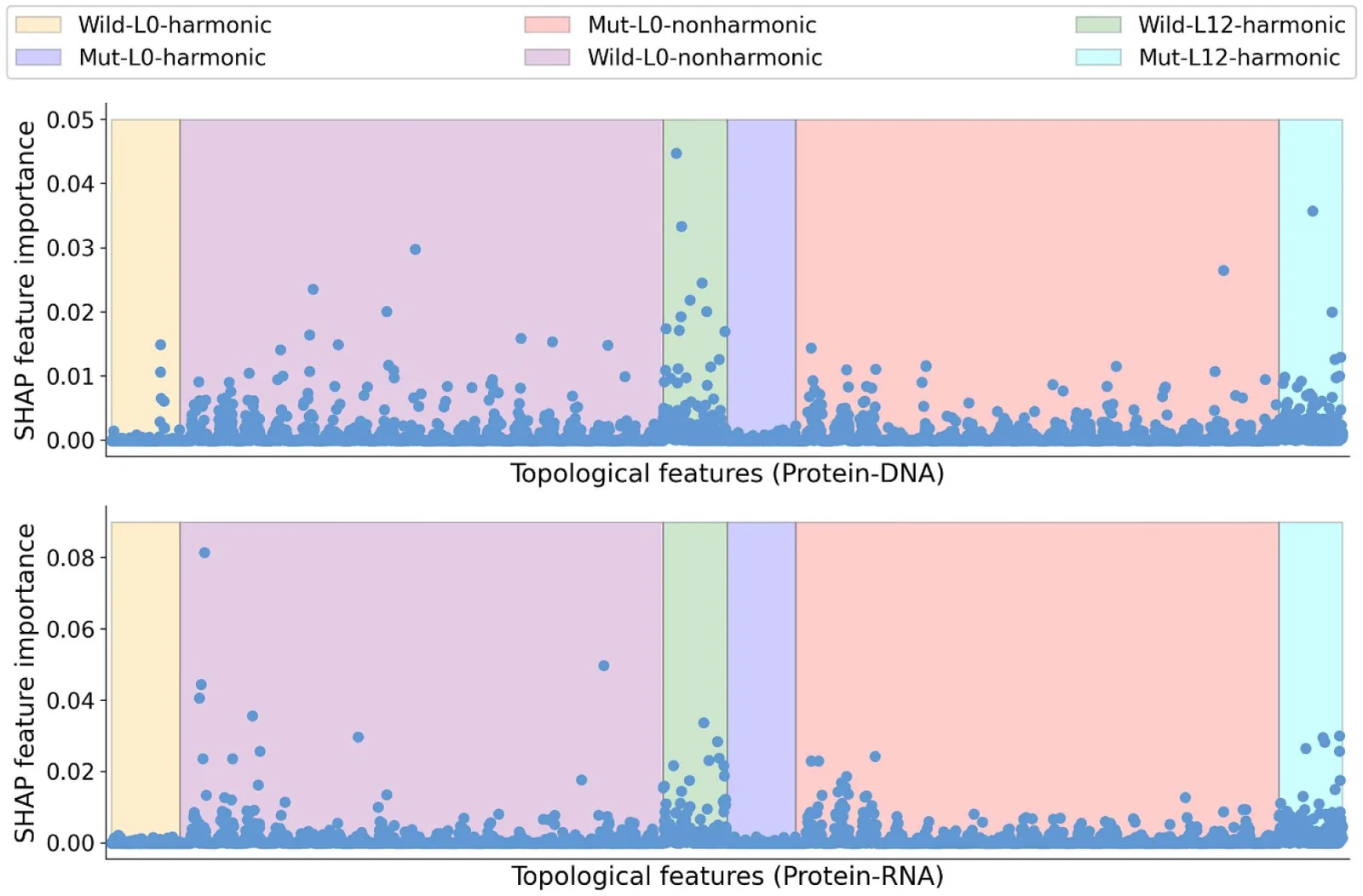
Illustration of SHAP-based feature importance analysis of the topological features.

To elucidate the contribution of topological features to the prediction tasks, we employed the Shapley Additive exPlanations (SHAPs) method [48] to assess the global importance of these features. The topological features comprise the harmonic and non-harmonic spectra of the zero-dimensional Laplacians, as well as the harmonic spectra of the one- and two-dimensional Laplacians. These spectral descriptors were derived from both the wild-type and mutant-type protein–nucleic acid complexes, detailed definitions and extraction procedures are provided in the ‘topological features’ section of the supplementary information. The results of the SHAP analysis are presented in figure 4. As shown, for protein–DNA prediction, the harmonic spectra of the one- and two-dimensional Laplacians contribute most significantly, suggesting that loop and void structures within the protein–DNA complex play critical roles in determining binding behavior. A similar trend is observed for protein–RNA prediction. Moreover, the non-harmonic spectra also exhibit contributions to model performance for both protein–DNA and protein–RNA predictions. Interestingly, the zero-dimensional harmonic spectra of the mutant type contribute least to protein–DNA prediction, and those of both the wild and mutant types contribute minimally to protein–RNA prediction. These findings collectively highlight the complementary importance of non-harmonic spectral information captured by the persistent Laplacians.

## 3. Conclusions

Studying the effects of protein mutations on protein–nucleic acid binding affinities is essential for understanding disease mechanisms and developing effective therapeutic strategies. However, experimental methods for measuring binding affinities are often time-consuming and labor-intensive, while existing computational approaches yield only moderate predictive performance. Therefore, it is imperative to explore novel methodologies that can improve prediction accuracy. In this work, we propose a TopoML model that leverages the persistent Laplacian from TDA to predict protein–nucleic acid binding affinity changes induced by amino acid mutations. By integrating features from multiple perspectives, including physicochemical properties, topological structures, and sequence embeddings derived from a pretrained protein Transformer, our model achieves a comprehensive and robust representation of protein–nucleic acid interactions. This integrative approach enables our model to outperform existing methods on both protein–DNA and protein–RNA datasets.

Our model can be further improved along the following lines: (1) Topological features: in the current study, we employ the standard persistent combinatorial Laplacian of simplicial complexes. However, recent developments have introduced more advanced mathematical tools, such as the persistent sheaf Laplacian [49] and the persistent Dirac operator [50, 51], which may provide deeper insights into the structure of protein–nucleic acid complexes. Exploring the applicability of these tools is a promising direction for future work. (2) Machine learning algorithms: while we use the gradient boosting tree for regression in this study, alternative algorithms such as random forests [52] and XGBoost [53] may also offer competitive performance due to the small data sizes. Additionally, the stacking ensemble strategies that combine multiple learning algorithms typically yield performance better than any single one of the models [54], and thus merit further investigation.

## 4. Materials and methods

Here, we describe three types of features extracted from protein–nucleic acid complexes. A summary of the software packages used in this study is provided in section 4 of the supplementary information, and the detailed machine learning parameters are listed in table 2 of the supplementary information.

### 4.1. Topological features

In TopoML, the topological features are derived from the persistent Laplacians. In the following, we give a brief introduction of the persistent Laplacians of simplicial complexes, details about how to use the persistent Laplacians to extract topological features can be found in section 2 of the supplementary information.

An abstract simplicial complex K over a vertex set V is a collection of non-empty subsets of V such that for every σ∈K and every nonempty subset τ⊂σ, we have τ∈K. An element σ∈K consisting of p+1 vertices is called a p-simplex. The dimension of a p-simplex is defined as p, and the dimension of a simplicial complex is defined as the maximal dimension of its simplices. Any graph can be regarded as a one-dimensional simplicial complex.

An oriented simplicial complex K is a simplicial complex equipped with an ordering of its vertex set. Each simplex of K inherits an orientation from the vertex ordering; a simplex together with this induced order is referred to as an oriented simplex. For simplicity, we refer to oriented simplices simply as simplices throughout the rest of this exposition.

We fix a field coefficient F. For a simplicial complex K, the pth chain group $C_{p}(K)$ is defined as the vector space over F with basis given by all the p-simplices in K. The boundary operator $\partial_{p}\;:C_{p}(K)\to C_{p-1}(K)$ is a linear map defined on a p-simplex $\sigma =[\nu_{0}\nu_{1}\cdots \nu_{p}]$ by

$$\partial \;(\sigma )=\sum_{i=0}^{p}{\left(-1\right)}^{i}\left[\nu_{0}\nu_{1}\cdots \nu_{i-1}\nu_{i+1}\cdots \nu_{p}\right].$$

These boundary operators satisfy the property $\partial_{p}\;\circ \;\partial_{p+1}=0$, forming a chain complex:

$$\cdots \to C_{p+1}(K)\overset{\;\partial_{p+1}\;}{\to }C_{p}(K)\overset{\;\partial_{p}\;}{\to }C_{p-1}(K)\overset{\;\partial_{p-1}\;}{\to }\cdots \overset{\;\partial_{1}\;}{\to }C_{0}(K)\to 0.$$

The pth Betti number, denoted $\beta_{p}$, is the rank of $H_{p}(K)$. Intuitively, $\beta_{0}$ counts the number of connected components, $\beta_{1}$ the number of loops, and $\beta_{2}$ the number of voids. These topological invariants capture essential structural information about the simplicial complex.

Consider the standard inner product on the chain groups, that is, for any two simplices σ and τ,

$$<\sigma ,\tau >=\delta_{\sigma ,\tau },$$

with $\delta_{\sigma ,\tau }$ being the Kronecker delta. Let $\partial_{p}^{*}$ denote the adjoint of $\partial_{p}$ under this inner product. The pth combinatorial Laplacian is then defined as

$$L_{p}={\left(\partial_{p}\right)}^{*}\partial_{p}+\partial_{p+1}{\left(\partial_{p+1}\right)}^{*}.$$

In matrix form, $\partial_{p}^{*}$ is the transpose of $\partial_{p}$. A fundamental property of the Laplacian is that the nullity of $L_{p}$ equals the pth Betti number $\beta_{p}$.

Now consider a filtration of the simplicial complex K, i.e. a nested sequence of simplicial complexes:

$$K_{0}\subset K_{1}\subset \cdots \subset K_{n}=K.$$

Applying the homology functor to this sequence yields a sequence of homology groups:

$$H_{p}(K_{0})\overset{\;i_{0}\;}{\to }H_{p}(K_{1})\overset{\;i_{1}\;}{\to }\cdots \overset{\;i_{n-1}\;}{\to }H_{p}(K_{n})\;,$$

where $i_{p}$ is the homomorphism induced by the inclusion map from $K_{p}$ to $K_{p+1}$. Let $i_{s,t}$ denote the composition $H_{p}(K_{s})\overset{\;i_{s}\;}{\to }H_{p}(K_{s+1})\overset{\;i_{s+1}\;}{\to }\cdots \overset{\;i_{t-1}\;}{\to }H_{p}(K_{t})$.

The pth persistent homology from $K_{s}$ to $K_{t}$ is defined as

$$H_{p}^{s,t}=Im(i_{s,t})\;,$$

which are the homology classes that appear at $K_{s}$ and are still alive at $K_{t}$.

Let $C_{p}^{s}=C_{p}(K_{s})$. Consider the subspace

$$C_{p}^{s,t}=\left\{\alpha \in C_{p}^{t}|\partial \;(\alpha )\in C_{p-1}^{s}\right\}.$$

Then we have

$$C_{p+1}^{s,t}\overset{\;\partial_{p+1}^{s,t}\;}{\to }C_{p}^{s}\overset{\;\partial_{p}\;}{\to }C_{p-1}^{s}$$

where $\partial_{p+1}^{s,t}$ is the restriction of $\partial_{p+1}$ on the subspace $C_{p+1}^{s,t}$.

The persistent Laplacian from $K_{s}$ to $K_{t}$ is defined as

$$L_{p}^{s,t}=\partial_{p+1}^{s,t}{\left(\partial_{p+1}^{s,t}\right)}^{*}+{\left(\partial_{p}\right)}^{*}\partial_{p}.$$

Notably, the nullity of $L_{p}^{s,t}$ is the p-persistent Betti number from $K_{s}$ to $K_{t}$,

$$\text{rank}\;(H_{p}^{s,t})=\text{rank}\;(L_{p}^{s,t}).$$

Consequently, the persistent homology information is fully encoded in the persistent Laplacian, which corresponds to its harmonic component.

**Figure 5.**
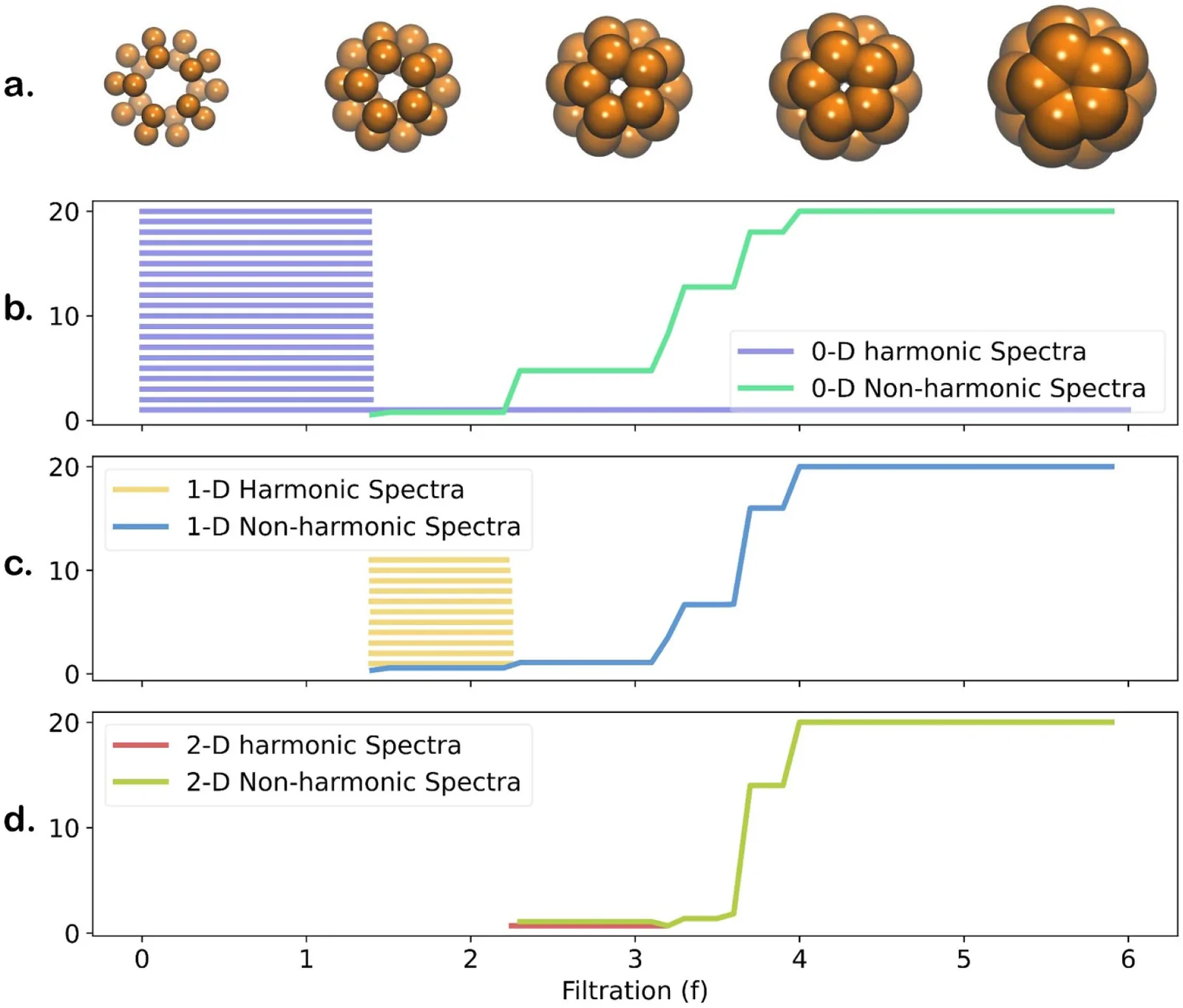
Illustration of persistent Laplacians. (a): a filtration process from the *C*_20_. Persistent Laplacian spectra in zero (b), one (c) and two (d) dimensions.

An example can be found in figure 5. As shown in the figure, a Vietori–Rips filtration of the *C*_20_ molecule is shown in (a), while (b)–(d) show the corresponding persistent Laplacian spectra in dimensions 0, 1, and 2, respectively. The harmonic spectra of the persistent Laplacian in (b)–(d) are isomorphic to persistent homology, capturing essential topological features such as connected components, loops, and voids. Specifically, in the zero-dimensional harmonic component, the 20 bars correspond to the 20 atoms in the *C*_20_ structure. In the one-dimensional harmonic part, the 11 bars represent the 12 pentagonal loops in the structure (with one loop topologically dependent on the others). In the two-dimensional case, there is a single bar corresponding to the global void enclosed by the *C*_20_ cage. These harmonic spectra efficiently characterize the multiscale topological information of the data. Beyond topological information, the non-harmonic spectra encode additional geometric information that persistent homology alone cannot capture. As shown in figures 5(b)-(d), the smallest non-zero eigenvalues of the persistent Laplacians are plotted for the 0-D, 1-D, and 2D cases, respectively. Notably, starting at a filtration value of approximately 3.2 Å, the persistent homology no longer changes, as no further homotopic changes occur. However, the continued addition of higher-dimensional simplices (edges, triangles, and tetrahedra) alters the simplicial complex induced by the geometric connectivity of the Laplacian model. These geometric variations, invisible to persistent homology, are effectively captured by the persistent Laplacian through its non-harmonic spectral components.

### 4.2. Physicochemical features

In addition to topological features, our model incorporates a set of physicochemical features at both the atom-level and the residue-level to comprehensively characterize each protein–nucleic acid complex. At the atom-level, the features include solvent-excluded surface area, partial atomic charges, Coulombic interaction energies, van der Waals interaction energies, and electrostatic solvation free energy [55-57]. At the residue-level, the features comprise amino acid composition in the neighborhood of the mutation site, p*K*_*a*_ shifts due to mutation, position-specific scoring matrix-based features, and secondary structure-based descriptors. These features collectively form a 783-dimensional vector representing the physicochemical profile of each protein–nucleic acid complex. Details can be found in section 3 of the supplementary information.

### 4.3. Sequence features

Protein large language models, such as evolutionary scale modeling (ESM) [58] and ProtTrans [59], which are trained on hundreds of millions of protein sequences, have demonstrated remarkable performance in various protein analysis tasks. Their effectiveness has been further enhanced through the application of hybrid fine-tuning strategies. In the TopoML model, we employ the ESM-2 Transformer architecture [60] to generate sequence embeddings. Specifically, we use the esm2_t33_650M_UR50D model, which is trained using self-supervised learning via masked language modeling on a comprehensive protein sequence database. The ESM-2 model comprises 33 Transformer layers and approximately 650 million parameters. Each layer encodes input tokens into 1280-dimensional vectors. For each input sequence, we extract the representations from the final layer and compute the average across the sequence length, resulting in a single 1280-dimensional vector. Sequence embeddings are obtained for both the wild-type and the mutant protein sequences, and these vectors are concatenated to form a final 2560-dimensional representation used as the sequence feature in our model. For more details on the pretrained ESM-2 model, please refer to the [60].

## Supplementary Material

SI

The supplementary information, available in the file SI.pdf, contains detailed descriptions of structural region type definitions, topological features, physicochemical features, software packages utilized, machine learning parameters, and detailed model performance on test sets S115 and MPR79 for protein-RNA interactions.

## Figures and Tables

**Table 1.** Performance comparison of different types of features. The unit for MAE is kcal mol^−1^. Topo: topological feature from persistent Laplacian, Phy: physicochemical feature, Seq: sequence feature by pretrained Transformer.

	Topo		Phy		Seq		Topo + Phy + Seq	
	PCC	MAE	PCC	MAE	PCC	MAE	PCC	MAE
Protein–DNA	0.648	0.65	0.638	0.654	0.608	0.657	0.681	0.612
Protein–RNA	0.678	0.825	0.67	0.843	0.678	0.817	0.721	0.785

## Data Availability

The data that support the findings of this study are openly available at the following URL/DOI: https://github.com/LiuXiangMath/TopoML. Supplementary data available at https://doi.org/10.1088/2632-2153/ae1714/data1.
