# Supplementary material for: Topological machine learning for protein–nucleic acid binding affinity changes upon mutation
Source: Mach Learn Sci Technol. Author manuscript; Available in PMC 2026 Aug 29. (PMC13523844; doi:10.1088/2632-2153/ae1714)
Supplement: Supplementary file 1 [file NIHMS2194918-supplement-SI.pdf]

Supplementary Information

Topological Machine Learning for Protein-Nucleic Acid Binding Affinity Changes Upon Mutation

Xiang Liu[1], Junjie Wee[1] and Guo-Wei Wei *[1,2,3]

[1] *Department of Mathematics, Michigan State University, MI, 48824, USA*
[2] *Department of Electrical and Computer Engineering, Michigan State University, MI 48824, USA*
[3] *Department of Biochemistry and Molecular Biology, Michigan State University, MI 48824, USA*

Contents

1 Structural Region Types **2**

2 Topological Features **2**

3 PhysicoChemical Features **3**

3.1 Atom-level Features 3

3.2 Residue-level Features 4

4 Software Packages Used **5**

5 Machine Learning Parameters **6**

6 Detailed Results **6**

*Corresponding author: weig@msu.edu

1 Structural Region Types

Table 1: Structural regions definition based on rASA, the definition is from [1]. rASAm: relative ASA in monomer; rASAc: relative ASA in complex; ΔrASA = rASAm − rASAc.

Region Type	$\Delta rASA$	rASAc	rASAm
Interior	0	<0.25	
Surface	0	>0.25	
Support	>0		<0.25
Rim	>0	>0.25	
Core	>0	<0.25	>0.25

2 Topological Features

In our TopoML model, each protein-nucleic acid complex is represented as a set of simplicial complexes and their persistent Laplacians are computed to extract topological features. Since protein-nucleic acid complexes are typically of large sizes, and their interactions primarily occur at the interface regions, we restrict our analysis to atoms near the binding and mutation sites in order to reduce computational cost. More specifically, for each protein-nucleic complex, the following four atom sets are considered:

- $\mathcal{A}_m$: atoms within the mutation site
- $\mathcal{A}_{mn}(r)$: atoms in the neighborhood of the mutation site within a cutoff of r
- $\mathcal{A}_p(r)$: protein atoms within a distance r from the binding site
- $\mathcal{A}_n(r)$: nucleic acid atoms within a distance r from the binding site

Both the wild-type and mutant protein structures are considered, and the element-specific representation [2] is used to capture pairwise interactions between different atom types. Specifically, the carbon (C), nitrogen (N), and oxygen (O) atoms in the mutation site and its neighborhood are used to form nine pairwise atom combinations. Similarly, the C, N, and O atoms in the protein and nucleic acid binding regions yield another nine combinations. In total, 36 atom sets are defined for each protein-nucleic acid complex. We construct both Vietoris-Rips complexes [3] and Alpha complexes [4] on these atom sets to model their topological interactions.

For Vietoris-Rips complex, the following distance d is used to model the interactions

$$d(x,y) = \begin{cases} E_d(x,y) & , S_x \neq S_y \\ \infty & , otherwise \end{cases} \tag{1}$$

where $E_d(x, y)$ denotes the Euclidean distance between atoms x and y, and S_x indicates the atom set to which x belongs. For example, in modeling interactions between the mutation site and its neighboring atoms, any pair of atoms within the mutation site itself are considered to be at infinite distance. For each Rips complex, we compute the zero-dimensional persistent Laplacian. The harmonic component is calculated over a filtration from 0 Å to 8 Å with a step size of 0.5 Å. A bin-counting method is applied to generate a 16-dimensional feature vector for each atom set. For the non-harmonic components, the same filtration is used, and seven statistical properties are extracted from the non-zero eigenvalue spectrum of the persistent Laplacian: maximum, minimum, mean, sum, standard deviation, variance, and count of eigenvalues. This yields a 112-dimensional feature vector per atom set.

For Alpha complex, the standard Euclidean distance is used for all 36 atom sets. In this case, we consider the one-dimensional and two-dimensional persistent Laplacians. To simplify computation, only the harmonic component is retained, which corresponds directly to persistent homology. Using barcode representations, we extract the following statistical features: (1) sum, maximum, and mean of bar lengths; (2) maximum and minimum of bar birth times; and (3) maximum and minimum of bar death times. This yields a 14-dimensional feature vector per atom set. In addition to the 36 atom sets, we also compute the same 14-dimensional Alpha complex features on two global atom sets: all heavy atoms in the wild-type and mutant complexes, respectively, to capture the global topological structural information. The total topological feature for a protein-nucleic complex is a $5140=(112+16)\times36+14\times38$ dimensional vector.

3 PhysicoChemical Features

In TopoML, in addition to topological and sequence features, we incorporate physico-chemical features to capture crucial chemical and physical properties for protein-nucleic acid interactions. These physicochemical features are divided into two major categories: atom-level and residue-level features. The inclusion of these features ensures a comprehensive and robust characterization of the protein-nucleic acid interactions. The total feature dimension for the physicochemical component is 783.

3.1 Atom-level Features

For atom-level features, the element-specific strategy is employed to categorize atoms into distinct sets. Specifically, for proteins, atoms are grouped into seven categories: carbon (C), nitrogen (N), oxygen (O), sulfur (S), hydrogen (H), heavy atoms, and all atoms. Similarly, for nucleic acids, atoms are grouped into carbon (C), nitrogen (N), oxygen (O), phosphorus (P), hydrogen (H), heavy atoms, and all atoms. These groups are further subdivided based on spatial regions, including the mutation region, the mutation neighborhood region, the protein binding region, the nucleic acid binding region, and the entire structure.

The atom-level features are generated based on these groups. The wild-type, mutant, and their difference are considered to give a comprehensive representation.

1. Surface Area: Atom-level solvent-excluded surface areas are calculated using ESES [5]. Atom surface areas within each group are summed up to generate one feature, leading to $(5 \times 7 + 1) \times 3 = 108$ features.

2. Partial Charge: Partial charges are computed using PDB2PQR [6] with the AMBER force field. For each atom group, both the sum of partial charges and the sum of absolute partial charges are considered. This leads to $5 \times 7 \times 3 \times 2 + 2 \times 3 = 216$ features.

3. Coulomb Interaction: The Coulomb energy of the i-th atom is computed as the sum of pairwise Coulomb interactions with every other atom according to the following formula:
$$C_i = \sum_{j \neq i} k_e \frac{q_i q_j}{r_{ij}}$$
where k_e is the Coulomb constant. The constant of 1 is used in our computation. q_i is the partial charge of the i-th atom, and r_{ij} is the Euclidean distance between i-th and j-th atoms. Only five atom element groups (C, N, O, S/P, and heavy atoms) are considered in the computation. Both the coulomb energy and its absolute value are computed. Totally, there are $4 \times 5 \times 2 \times 3 + 6 \times 2 \times 3 = 156$ features.

4. Van der Waals interaction. The van der Waals energy of the i-th atom is computed as the sum of pairwise Lennard-Jones potentials with all other atoms.
$$V_i = \sum_{j \neq i} \epsilon[(\frac{r_i + r_j}{r_{ij}})^{12} - 2(\frac{r_i + r_j}{r_{ij}})^6]$$
where r_i is the atom radius of i-th atom and ϵ is the depth of potential well. We use $\epsilon = 1$ in the computation. Similarly, only 5 atom element groups (C, N, O, S/P, and heavy atoms) are considered. The feature size is $4 \times 5 \times 3 + 6 \times 3 = 78$.

5. Electrostatic Solvation Free Energy. The electrostatic solvation free energy for each atom is computed using the Poisson-Boltzmann model through the software MIBPM [7]. By summing up all solvation free energies within each group, $5 \times 7 \times 3 + 3 = 108$ features are generated.

3.2 Residue-level Features

1. Mutation Neighborhood Amino Acid Composition. Neighboring residues within 12 Å of the mutation site are considered. These residues are classified into six categories: hydrophobic, polar, polar uncharged, positively charged, negatively charged, and special cases. Both the count and percentage for each category are used as environmental

features. Additionally, the sum, mean, and variance of residue volumes, surface areas, molecular weights, and hydropathy scores are calculated, along with the sum of charges. There are a total of $6 \times 2 + 3 \times 4 + 1 = 25$ features.

2. pKa shifts. The pKa values of residues are computed using PROPKA software [8]. Specifically, the seven ionizable amino acids are considered: ASP, GLU, ARG, LYS, HIS, CYS, and TYR. The pKa values of the mutation site, N-terminal, and C-terminal residues are considered for both wild-type and mutant types. The maximum, minimum, and sum of the differences between the wild-type and mutant pKa values are computed, along with the minimum and sum of their absolute differences. Additionally, the sum and absolute sum of pKa values within each group are included. This results in $3 \times 2 + 3 + 2 + 7 \times 2 = 25$ features.

3. Position Specific Scoring Matrix. Features are computed from the conservation scores in the PSSM of the mutation site. The conservation scores are computed by PSI-BLAST [9]. The feature size is $8 \times 2 = 16$ for wild-type and mutant proteins.

4. Secondary Structure. The SPIDER software [10] is used to compute the probability of residue torsion angle and a residue being in a coil, alpha helix, and beta strand. The features are computed for wild-typ, mutant type, and their difference, resulting in $17 \times 3 = 51$ features.

4 Software Packages Used

- The Jackal software [11] is used to generate mutant protein-nucleic acid complex.
- The PDB2PQR [6] with the AMBER force field is used to generate the partial charges of atoms.
- The ESES software [5] is used to compute the solvent excluded surface area of atoms.
- The MIBPB [7] is used to compute the electrostatic solvation free energy of atoms.
- The PROPKA package [8] is used to compute the pka values.
- The PSI-BLAST software [9] with an uniref50 database is used to compute the PSSM.
- The SPIDER software [10] is used to compute the secondary structure features.
- The GUDHI [12] and scipy [13] packages are used to compute the persistent Laplacian features.
- The ESM-2 [14] is used to generate the sequence features.

5 Machine Learning Parameters

Table 2: Parameters for gradient boosting tree

No. of estimators	10000
max_depth	7
min_sample_split	3
learning_rate	1e-3
max_features	sqrt
subsample	0.5

6 Detailed Results

Table 3: Performance comparison of TopoML with other models in a test set of 115 mutants for protein-RNA interactions. The benchmark results are taken from the literature [15]. The unit for MAE is kcal/mol. The best results are in bold. Our results are reported as the mean and standard deviation over 100 independent runs.

Models	PCC	MAE
FoldX	0.48	1.93
mCSM-NA	0.13	1.69
PremPR	0.26	1.58
PRA-MutPred	0.67	0.81
**TopoML**	$\mathbf{0.72}_{0.003}$	$\mathbf{0.77}_{0.004}$

Table 4: Performance comparison of TopoML with other models in the MPR79 test set for protein-RNA interactions. The benchmark results are taken from the literature [15]. The unit for RMSE is kcal/mol. The best results are in bold. Our results are reported as the mean and standard deviation over 100 independent runs.

Models	PCC	RMSE
PEMPNI	0.407	0.731
PNABCE	0.417	0.728
PRA-MutPred	0.50	0.72
**TopoML**	$\mathbf{0.53}_{0.004}$	$\mathbf{0.70}_{0.004}$

References

[1] Emmanuel D Levy. A simple definition of structural regions in proteins and its use in analyzing interface evolution. *Journal of molecular biology*, 403(4):660–670, 2010.

[2] Zixuan Cang and Guo-Wei Wei. Integration of element specific persistent homology and machine learning for protein-ligand binding affinity prediction. *International journal for numerical methods in biomedical engineering*, 34(2):e2914, 2018.

[3] Leopold Vietoris. Über den höheren zusammenhang kompakter räume und eine klasse von zusammenhangstreuen abbildungen. *Mathematische Annalen*, 97(1):454–472, 1927.

[4] Herbert Edelsbrunner. Alpha shapes-a survey. In *Tessellations in the sciences: Virtues, techniques and applications of geometric tilings*. 2011.

[5] Beibei Liu, Bao Wang, Rundong Zhao, Yiying Tong, and Guo-Wei Wei. ESES: Software for Eulerian solvent excluded surface, 2017.

[6] Todd J Dolinsky, Jens E Nielsen, J Andrew McCammon, and Nathan A Baker. Pdb2pqr: an automated pipeline for the setup of poisson–boltzmann electrostatics calculations. *Nucleic acids research*, 32(suppl_2):W665–W667, 2004.

[7] Duan Chen, Zhan Chen, Changjun Chen, Weihua Geng, and Guo-Wei Wei. MIBPB: a software package for electrostatic analysis. *Journal of computational chemistry*, 32(4): 756–770, 2011.

[8] Delphine C Bas, David M Rogers, and Jan H Jensen. Very fast prediction and rationalization of pka values for protein–ligand complexes. *Proteins: Structure, Function, and Bioinformatics*, 73(3):765–783, 2008.

[9] Stephen F Altschul, Thomas L Madden, Alejandro A Schäffer, Jinghui Zhang, Zheng Zhang, Webb Miller, and David J Lipman. Gapped blast and psi-blast: a new generation of protein database search programs. *Nucleic acids research*, 25(17):3389–3402, 1997.

[10] Yuedong Yang, Rhys Heffernan, Kuldip Paliwal, James Lyons, Abdollah Dehzangi, Alok Sharma, Jihua Wang, Abdul Sattar, and Yaoqi Zhou. Spider2: a package to predict secondary structure, accessible surface area, and main-chain torsional angles by deep neural networks. *Prediction of protein secondary structure*, pages 55–63, 2017.

[11] Jason Z Xiang and B Honig. Jackal: A protein structure modeling package. *Columbia University and Howard Hughes Medical Institute, New York*, 2002.

[12] Clément Maria, Jean-Daniel Boissonnat, Marc Glisse, and Mariette Yvinec. The gudhi library: Simplicial complexes and persistent homology. In *Mathematical Software–ICMS 2014: 4th International Congress, Seoul, South Korea, August 5-9, 2014. Proceedings 4*, pages 167–174. Springer, 2014.

[13] Ralf Gommers, Pauli Virtanen, Matt Haberland, Evgeni Burovski, Tyler Reddy, Warren Weckesser, Travis E Oliphant, David Cournapeau, Andrew Nelson, Pamphile Roy, et al. scipy/scipy: Scipy 1.15. 0. *Zenodo*, 2024.

[14] Zeming Lin, Halil Akin, Roshan Rao, Brian Hie, Zhongkai Zhu, Wenting Lu, Nikita Smetanin, Robert Verkuil, Ori Kabeli, Yaniv Shmueli, et al. Evolutionary-scale prediction of atomic-level protein structure with a language model. *Science*, 379(6637): 1123–1130, 2023.

[15] K Harini, M Sekijima, and M Michael Gromiha. Pra-mutpred: Predicting the effect of point mutations in protein–rna complexes using structural features. *Journal of Chemical Information and Modeling*, 2025.
